# YAP and TAZ in Lung Cancer: Oncogenic Role and Clinical Targeting

**DOI:** 10.3390/cancers10050137

**Published:** 2018-05-06

**Authors:** Federica Lo Sardo, Sabrina Strano, Giovanni Blandino

**Affiliations:** 1 Oncogenomic and Epigenetic Unit, Molecular Medicine Area Regina Elena National Cancer Institute, via Elio Chianesi 53, 00144 Rome, Italy; federica.losardo@ifo.gov.it; 2 Molecular Chemoprevention Group, Molecular Medicine Area Regina Elena National Cancer Institute, via Elio Chianesi 53 00144 Rome, Italy; sabrina.strano@ifo.gov.it

**Keywords:** YAP/TAZ, lung cancer, NSCLC, therapeutic targets

## Abstract

Lung cancer is the leading cause of cancer death in the world and there is no current treatment able to efficiently treat the disease as the tumor is often diagnosed at an advanced stage. Moreover, cancer cells are often resistant or acquire resistance to the treatment. Further knowledge of the mechanisms driving lung tumorigenesis, aggressiveness, metastasization, and resistance to treatments could provide new tools for detecting the disease at an earlier stage and for a better response to therapy. In this scenario, Yes Associated Protein (YAP) and Trascriptional Coactivator with PDZ-binding motif (TAZ), the final effectors of the Hippo signaling transduction pathway, are emerging as promising therapeutic targets. Here, we will discuss the most recent advances made in YAP and TAZ biology in lung cancer and, more importantly, on the newly discovered mechanisms of YAP and TAZ inhibition in lung cancer as well as their clinical implications.

## 1. Introduction

Among solid tumors, lung cancer is the first cause of cancer death worldwide with a 5-year survival rate lower than 20%. Nearly 80–85% of lung cancers are non-small cell lung cancer (NSCLC), which include adenocarcinoma (LAC), lung squamous cell carcinoma (LSCC), and large-cell carcinoma [1]. One of the reasons for this short survival is the lack of clear symptoms occurring until advanced stages of the disease. Many diagnoses are given when the cancer has already progressed beyond a localized state. Moreover, in some patients, cancer cells are often resistant or acquire resistance to therapy. Finally, in 10% to 25% of lung cancer patients, brain metastases can occur and are associated with unfavorable prognosis and loss of cognitive functions [2]. Up until now, current treatment based on surgery, radiation, chemotherapy, laser therapy, and photodynamic therapy associated with palliative care only increase the overall survival and quality of life of patients, but NSCLC still remains one of the most aggressive malignant tumors with the lowest survival rate [3,4,5]. This observation spurred the development of new technology for the early detection of lung cancer in people who are at very high risk [6,7,8] as well as the development of targeted therapies for patients with known mutations driving lung cancer. In this scenario, the characterization of early molecular biomarkers can provide a useful tool for early diagnosis while a deeper understanding of the mechanisms driving lung transformation, metastasization, and resistance to therapy could provide new therapeutic targets.

## 2. The Hippo Pathway

The Hippo signaling transduction pathway controls animal organ development, growth, and regeneration upon injury, and its dysregulation is often involved in tumorigenesis [9,10,11]. Cell contact, cell polarity, and metabolic and mechanical signals, which change during organ development and growth to properly orchestrate these processes, regulate the activity of the Hippo pathway core components, consisting of a cascade of kinases (MST1/2 and LATS1/2) with adaptor proteins (SAV and MOB) whose final targets are the transcriptional coactivators Yes Associated Protein (YAP) and Trascriptional Coactivator with PDZ-binding motif (TAZ) [11,12,13]. When the Hippo pathway is turned on, YAP and TAZ are phosphorylated by LATS1/2, promoting their cytoplasmic sequestration and proteasome-mediated degradation. When the Hippo pathway is turned off, YAP and TAZ are dephosphorylated and translocated to the nucleus where they are able to activate target genes in association with different transcription factors ultimately regulating cell growth, metabolism, proliferation, migration, invasion, or cell death [12,14,15]. Recent evidence added new complexity to this simple model, adding new players and regulators to the Hippo pathway core kinases [16]. For example, two groups of MAP4Ks (mitogen-activated protein kinase)—MAP4K1/2/3/5 and MAP4K4/6/8—were recently discovered acting in parallel with MST1/2 to phosphorylate and activate LATS1/2 [17,18]. Moreover, Hippo core kinases may be involved in the regulation of proliferation and the cell cycle independently of YAP/TAZ function [19,20,21] and, vice versa, YAP and TAZ are not exclusively regulated by the Hippo pathway core kinases. In fact, YAP and TAZ may undergo different post-translational modifications beyond Ser127 phosphorylation and can interact with several different protein partners, resulting in different activation states and/or subcellular localization of YAP/TAZ [22,23,24,25,26,27,28,29,30]. Additionally, YAP/TAZ and other Hippo pathway components crosstalk with several other signaling pathways, such as EGFR, Wnt, TGF-β, and Notch, involved in development and cell proliferation (reviewed in [31,32]). Finally, YAP and TAZ are often treated as single proteins, but they are two distinct proteins which exert both overlapping and exclusive functions [9,33,34,35,36].

## 3. Regulation of the Hippo Pathway

Physiological organ development and growth or regeneration upon injury are the final results of specific patterns of cell growth, proliferation, migration, commitment, differentiation, senescence, or apoptosis. These, in turn, are orchestrated by the mechanical and biochemical stimuli originating from intrinsic cell machineries and from the extracellular matrix (ECM). These stimuli regulate proteins involved in mechanotransduction, cell junction, cell polarity, G-protein-coupled receptor (GPCR) signaling, receptor tyrosine kinase (RTK) mitogenic signaling, and metabolism which, in turn, regulate the core Hippo pathway and subsequent YAP and TAZ nuclear activity [11,12,13]. In general, low mechanical stress (which can be experienced by cells grown on a soft extracellular substrate or at a high cell density), serum deprivation, low glucose and nutrients, as well as diffusible signals that inhibit cell proliferation and metabolism are activators of the Hippo pathway core kinases and thus inhibit YAP/TAZ nuclear activity. Conversely, low cell density, stiff extracellular substrate or high mechanical stress transduced to the cytoskeleton, mitogenic signals, inflammation, and high nutrient uptake activate nuclear YAP and TAZ [11,12]. The nature and the position of the post-translational modifications of YAP and/or TAZ, their subcellular localization, their total abundance, their specific interactions with other proteins, and their crosstalk with other signaling pathways will eventually determine their final biological outcome. This, in turn, depends on (1) the signals coming from the extracellular environment (upstream signaling) and (2) the cell type which transduces these signals (downstream signaling). 

## 4. YAP and TAZ are Pro-Oncogenic in Solid Tumors

During physiologic embryogenesis and development, YAP and TAZ cooperate with transcriptional complexes in the regulation of cell stemness, cell growth, specification and differentiation, while in the adult, YAP and TAZ are required for proliferation of adult progenitors/stem cells and for the regeneration of damaged tissues [37,38]. These functions are elicited through the regulation of different transcriptional programs in a context-dependent manner. The dysregulation of YAP and TAZ function in adult cells has shown both protumorigenic and pro-apoptotic effects in different experimental systems and conditions. 

Generally, in hematologic malignancies, YAP is either deleted or downregulated and lower YAP expression correlates with poorer prognosis and shorter survival of patients [39]. In contrast, in many solid cancers, YAP and TAZ behave as oncogenes and are upregulated or hyperactivated compared to normal tissues, and a higher YAP/TAZ level or activity correlates with poorer prognosis and shorter patient survival [9,10,11]. 

**Figure 1:**
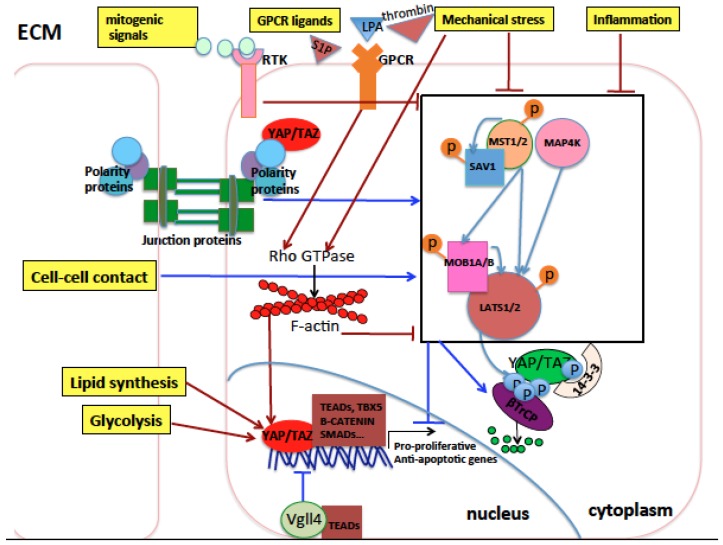
Schematic representation of the Hippo pathway core components and their upstream regulators in mammals. The extracellular matrix (ECM), the cytoplasm, and the nucleus of cells are represented. Arrows indicate activation, while blunt lines indicate inhibition. Activation indicates an increase in protein levels or activity, while inhibition indicates a decrease in protein levels or activity. Red indicates eventual activation of nuclear Yes Associated Protein (YAP)/Trascriptional Coactivator with PDZ-binding motif (TAZ), while dark blue indicates eventual inhibition of nuclear YAP/TAZ. Light blue arrows indicate phosphorylation of proteins by kinases. Orange or light blue balls indicate phosphorylation sites of target proteins. The Hippo core kinase cassette is represented inside a black rectangle. Cell polarity and cell junction proteins activate Hippo pathway core kinases and inhibit YAP/TAZ nuclear function. Conversely, G-protein-coupled receptor (GPCR) signaling, mitogenic signals, inflammation, and mechanical stress coming from the ECM inhibit the Hippo pathway core kinases through mechanisms either dependent on or independent of the Rho GTPase signaling which in turn stabilizes the actin cytoskeleton core kinases thereby activating YAP/TAZ nuclear activity. Lipid biosynthesis and glycolysis activate nuclear YAP and TAZ. In the nucleus, YAP and TAZ interact with different transcription factors and the resulting transcriptional outcome is context-specific, depending on the incoming signals to which cells are exposed and the cell type subjected to such signals.

In the context of solid cancers, YAP and TAZ transcriptionally activate genes involved in cell pluripotency and stemness, proliferation, migration, and invasiveness, and associate with oncogenic transcription factors like TEAD1-4, Smad1, 2/3, KLF5, AP-1, β-catenin, TBX5, ERG, Erb-B4 and mutp53 [29,40,41,42,43,44,45,46,47,48,49,50,51]. Recent lines of evidence have shown YAP/TAZ’s role also in transcriptional repression of tumor suppressors [52]. Frequently, higher YAP/TAZ expression levels can be observed in tumoral compared to matched nontumoral tissues in different cancer types. However, increased activity of YAP and/or TAZ in solid cancer is rarely caused by genomic amplification of YAP/TAZ loci. Instead, the main mechanisms that increase YAP and TAZ oncogenic activity in vivo are the modifications of the extracellular and intracellular signals occurring during tumorigenesis, for example, the formation of a hypoxic environment, the disruption of the apico-basal cell polarization in epithelial tissues, the presence of inflammation and cell/tissue damage signals, and increased mechanical stress induced by a higher production of cellular and extracellular constituents by cancer cells [37,53,54,55,56]. Additionally, several metabolic alterations occurring in most tumors increase YAP/TAZ oncogenic function. For example, cancer cells preferentially metabolize glucose through glycolysis, producing less energy (ATP) but promoting anabolism, cell growth, and proliferation [57] compared to normal cells which preferentially use oxidative phosphorylation to produce ATP. Glycolysis has been shown to activate and sustain the protumorigenic functions of YAP and TAZ [58,59,60]. Also, the increase of lipid biosynthesis is required to sustain the increased tissue growth during tumorigenesis [61,62]. Among the pathways involved in lipid biosynthesis, the mevalonate pathway and the synthesis of monounsaturated fatty acids have been shown to sustain YAP/TAZ oncogenic function [63,64,65] and will be discussed later in detail. All these mechanisms are described in Figure 1. 

## 5. Hippo Pathway in Lung Cancer 

In physiological conditions, YAP and TAZ are required for normal lung development and regeneration after injury [66,67]. In a mouse model of developing lung, the apical polarity protein Crb3 inhibits YAP/TAZ nuclear activity by sequestering them at the apical plasma membrane together with LATS1/2 in proximal airway epithelial cells. This inhibits proliferation and promotes differentiation. Conversely, in undifferentiated basal progenitor cells, where Crb3 levels are undetectable, YAP/TAZ are mainly nuclear and promote cell stemness and proliferation. Downregulation of Crb3 in proximal airway epithelium increases YAP/TAZ nuclear localization and cellular proliferation, leading to morphological changes which resemble a premalignant epithelium [68]. This suggests that the subcellular localization of YAP and TAZ is fine-tuned during lung development, when a balance between nuclear and cytoplasmic YAP and TAZ is necessary to support normal organ development and patterning. 

Interestingly, in adults, YAP and TAZ are not required for normal tissue homeostasis, while numerous experimental results show that nuclear YAP and TAZ are required to drive lung tumor formation, survival, stemness, progression, metastasization, and resistance to therapy, in particular in non-small cell lung cancer (NSCLC) that will be thoroughly described in the following sections. Conversely and interestingly, in a specific subset of small cell lung cancer cell lines and patients, the loss of YAP1 has been shown to be associated with an increase of neuroendocrine markers and a poorer prognosis [69,70] similar to what has been observed in haematologic malignancies. However, much of the literature reports evidence of the oncogenic role of YAP and TAZ in NSCLC which will be the focus of our review; meanwhile, we will keep in mind the opposite role of YAP and TAZ observed in SCLC compared to NSCLC that become clinically relevant when NSCLC is transformed into SCLC and needs different therapeutic treatments [71].

In NSCLC, YAP overexpression has been associated with development, progression, and poor prognosis [72,73,74,75,76,77] similar to TAZ [78,79,80], and an epidemiological study showed that a germline YAP mutation resulting in its oncogenic hyperactivation is associated with the occurrence of lung cancer [81]. In vivo studies in mouse models of lung adenocarcinoma showed that the genetic loss of YAP reduces the number of tumor masses experimentally induced in mice, while the knock-down of YAP or TAZ in human NSCLC cells impairs tumor formation after injection into nude mice [82,83]. TAZ overexpression in normal bronchial epithelial cells was found to be sufficient to promote tumor formation when injected into nude mice in one study [80] and not in another study which used a different cell line [78], while overexpression of YAP alone was not able to induce de novo tumor formation but only to promote progression of tumors driven by Kirsten RAt Sarcoma (KRAS) or Liver Kinase B1 (LKB1) mutations in mice [82,83]. Moreover, YAP and TAZ staining was stronger in metastatic versus nonmetastatic experimentally induced tumors in mice [82,83,84].

When translating these results into clinics, an increase of the YAP protein was observed in the nucleus and not in the cytoplasm in lung cancer specimens compared to the matched normal tissues [76]. In another study, the presence of higher levels of nuclear YAP was associated with poorer prognosis while, conversely, higher levels of cytoplasmic YAP were associated with lower histologic grade and TNM stage in NSCLC [75]. This indicates that in different cellular compartments, YAP and/or TAZ may exert completely different functions. In particular, this study clearly reinforces the idea that the oncogenic function of YAP and TAZ is exerted mainly in the nucleus through the transcriptional regulation of genes involved in tumorigenesis, while in the cytoplasm, YAP and TAZ are prevented from activating pro-proliferative genes. In agreement with this hypothesis, in different cohorts of lung cancer patients, higher expression of genes belonging to the YAP/TAZ signature correlates with poorer prognosis [80,82].

The increase of YAP and TAZ nuclear activity can be induced by an increase of signals, receptors, or transducers which positively regulate YAP/TAZ activity or by a decrease in signals or molecules that negatively regulate YAP/TAZ oncogenic function. This depends on one hand on the acquired genetic and epigenetic alterations of the genes coding for YAP/TAZ regulators in cancer cells, and on the other hand on the mechanical and biochemical changes occurring in the tumor microenvironment to sustain tumor growth. For example, the oncogenic ABL1 and 2 kinases, shown to be overexpressed or hyperactivated in NSCLC [85,86,87,88] and involved in tumor growth and metastasization in experimental models of lung cancer [27,87,88], have been recently demonstrated to act in part through the activation of TAZ and β-catenin by inhibiting their binding to β-TrCP, thus increasing their stability. In mouse models, ABL1 and 2 inactivation reduced the metastasization of NSCLC to the bone and brain, impinging on the expression of a subset of TAZ/β-catenin oncogenic target genes. In clinical lung cancer patient data, high expression levels of ABL1 and ABL2 and concomitant high transcription of TAZ/β-catenin targets correlate with shorter survival [27]. Also, several RTKs including EGFR or their downstream transducers/effectors like Src/Ras/Raf/MEK and PI3K/AKT/mTOR are hyperactivated and contribute to bad prognosis in lung cancer [89,90,91,92,93,94,95,96]. EGFR signaling crosstalks with Wnt-βcatenin signaling [97], GPCR signaling [98,99], and Hippo signaling [100]. It participates in the positive regulation of YAP and TAZ oncogenic function in lung cancer [101] and can synergize with YAP and TAZ in lung tumorigenesis and tumor growth and invasion [102].

Among YAP/TAZ endogenous inhibitors, it has been shown that LATS2 is mutated or more frequently downregulated in NSCLC through multiple mechanisms such as promoter hypermethylation, or recruitment of repressive epigenetic complexes including long noncoding RNAs and repressive Enhancer of Zeste Homolog 2 (EZH2) [103,104,105,106,107]. Higher LATS2 levels in lung cancer patients contribute to better prognosis [108] while forced overexpression of LATS2 in vitro reduces tumorigenicity of NSCLC [104]. Similarly, LATS1 is downregulated in 60% of NSCLC cancers while its high level contributes to good prognosis and negatively regulates oncogenic YAP in NSCLC [105,109,110]. AMOT, a scaffold protein that sequesters YAP and TAZ into the cytoplasm, inhibiting their nuclear function, has been shown to decrease lung cancer progression in vitro while its knock-down increases lung cancer growth and metastasis in vivo, and is significantly reduced in lung cancer specimens [111]. Also, MST1 kinase has been shown to inhibit NSCLC growth in vitro and in vivo [112]. Moreover, VGLL4, an inhibitor of YAP–TEAD interaction, inhibits cancer progression through YAP inactivation and is downregulated in lung adenocarcinoma [113]. Ras-association domain family 1 isoform A (RASSF1A), which activates MST1/2 and LATS1 in the presence of DNA damage or other stress signals [114,115,116], is epigenetically inactivated on its promoter in 40% of primary NSCLC [117,118,119]. Importantly, a significant hypermethylation as well as somatic loss of etherozigosity (LOH) of the RASSF1A locus has been observed also in SCLC [118,119]. This inactivation is associated with poor prognosis [119,120]. Mechanistically, it has been shown that RASSF1A inactivation in normal bronchial epithelial cells contributes to cell invasion and migration in part through the activation of nuclear YAP [121]. LKB1 has been recently shown to suppress YAP/TAZ nuclear function through either MST1 activation [84] or Hippo-independent pathways [122] in response to metabolic stress. LKB1 inactivation is involved in tumorigenesis [123], it is the third most frequently mutated gene in adenocarcinoma [124], and somatic mutations of LKB1 have been reported in 20–30% of NSCLC and derivative cell lines [124,125,126,127,128]. Mutations in LKB1 gene often occur together with *KRAS*-activating mutations, the most frequent oncogenic mutations found in NSCLC [128]. In mouse lung cancer models, LKB1 downregulation does not drive tumorigenesis by itself but it induces a more aggressive phenotype in KRAS-driven tumors, suggesting a role in lung cancer progression and metastasis. Accordingly, in NSCLC cell lines, LKB1 represses the expression of pro-metastatic genes [126] or pro-survival genes [83], and its lower expression in patients correlates with advanced disease [128]. A recent study showed that the interference of RhoA, which transduces mechanical cytoskeletal forces to YAP/TAZ, inhibited proliferation and metastasization in a cellular model of NSCLC [129]. A recent study found that Ski inhibits YAP and mainly TAZ in lung because it induces their phosphorylation mediated by LATS1/2, thus inducing their proteasome-mediated degradation but not their cytoplasmic translocation. In 56% of NSCLC tissues, the Ski promoter is hypermethylated and its downregulation is associated with poorer prognosis [130]. 

A recent study by Noto and coworkers showed that YAP and TAZ are required for the maintenance of stemness properties in lung cancer cell cultures [64]. Before this report, the role of YAP and TAZ in cancer stem cells was investigated in detail only in breast cancer, hepatocarcinoma, and esophageal cancer [35,131,132,133]. Moreover, the authors showed that stearoyl-CoA-desaturase 1 (SCD1), involved in the conversion of saturated into monounsaturated fatty acids, is important for the expression, nuclear localization, and transcriptional activity of YAP and TAZ, in part by acting through the Wnt-β-catenin pathway. Accordingly, the high co-expression of SCD1, β-catenin, and YAP/TAZ has a negative prognostic value in adenocarcinoma lung cancer patients [64]. This is the first report showing a correlation between an increased biosynthetic lipid metabolism driven by tumorigenesis and a hyperactivation of oncogenic YAP/TAZ in NSCLC. Before this study, this had been described only in other cancer types [134]. This report couples with our recent report showing that pharmacological inhibition of the mevalonate pathway inhibits YAP/TAZ oncogenic function also in NSCLC models [135] (see below). Later on, another work confirmed a role for TAZ in promoting cell stemness and tumorigenesis in lung through the upregulation of ALDH1A [51]. 

**Figure 2:**
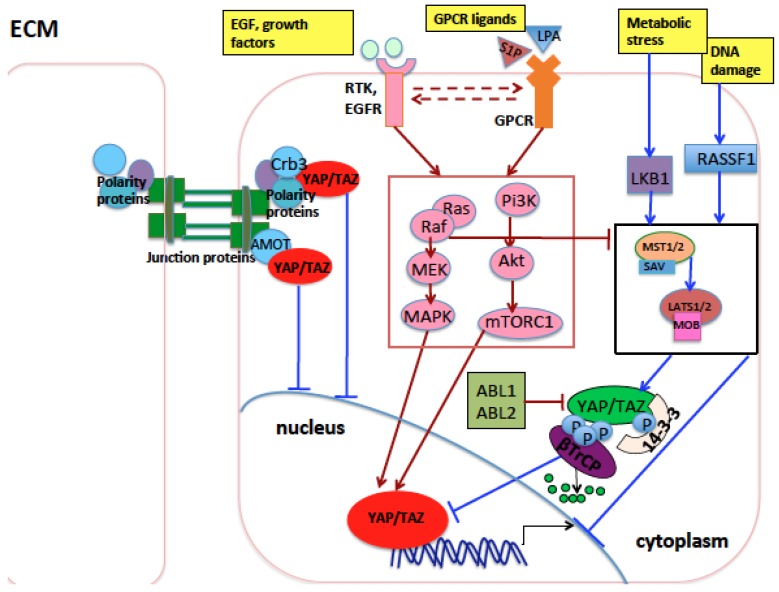
Main proteins and pathways that influence YAP and TAZ in lung development and tumorigenesis. During lung development, the Crb3 polarity protein sequesters YAP and TAZ at the apical plasma membrane preventing their oncogenic function in the nucleus. Amot, a component of the tight junction complexes, exerts a similar function. GPCR receptors, EGFR receptor, and other RTKs, when activated by ligands, induce the RAS/RAF/MEK/ERK and the PI3K/AKT/mTOR pathways (inside a red rectangle), which, in turn, activate oncogenic YAP and TAZ through mechanisms either dependent on or independent of Hippo pathway core kinases (inside black rectangle). Upon DNA damage, cell contact, or other stress stimuli, RASSF1A activates the MST1/2-LATS1/2 kinase cassete, inhibiting YAP and TAZ oncogenic function. In the nucleus, Vgll4 inhibits the oncogenic role of YAP and TAZ through impairing YAP and TAZ binding with TEAD transcription factors. Arrows indicate activation of the indicated proteins, while blunt lines indicate repression of targeted proteins. Lines or arrows in red indicate signals which eventually activate nuclear YAP/TAZ, while lines or arrows in blue indicate signals or proteins which eventually inhibit nuclear YAP/TAZ, through mechanisms either dependent on or independent of Hippo pathway core kinases. Dashed arrows indicate reciprocal crosstalk. Abbreviations: MAPK, mitogen-activated protein kinase; RTK: receptor tyrosine kinase; EGF: epidermal growth factor; EGFR: EGF receptor; GPCR: G-protein-coupled receptor.

YAP overexpression has been associated with increased resistance of NSCLC to current therapies because YAP provides parallel survival input to treatments [136,137,138]. All these observations underlie an important oncogenic role of YAP/TAZ as well as their upstream regulators in lung cancer (Table 1 and Figure 2). 

## 6. YAP and mutP53 Can Synergize in Lung Cancer

**Figure 3:**
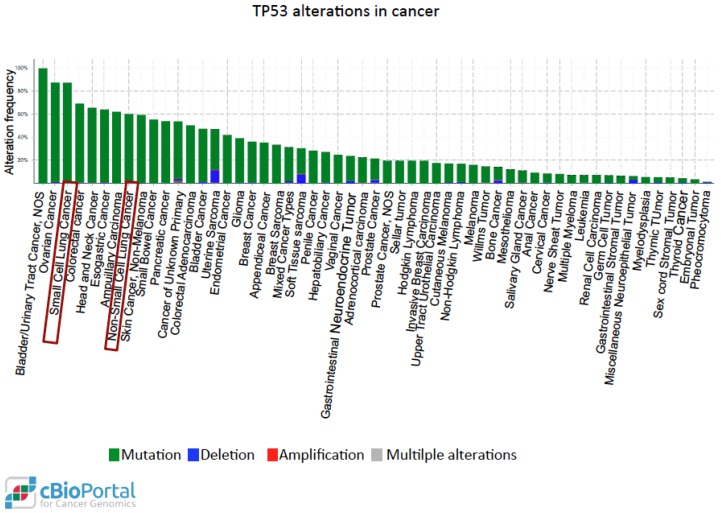
Distribution of the genetic alterations of the TP53 gene in human cancer. Data obtained from the cBioPortal for Cancer Genomics website [149] after querying for TP53 alterations in 54,483 samples from 215 studies of different cancer types. Each column represents the alteration frequency of the TP53 gene in each cancer type indicated. Sometimes the same cancer type is indicated more than once because it has been analyzed in different independent studies. Red boxes indicate lung cancer.

P53 is one of the most studied tumor suppressors and its coding gene, TP53, is the most commonly mutated in human cancer [139]. More than 50% of tumors arise from mutations of the wild-type TP53 gene sequence. These mutational events may lead to loss of tumor suppressor function of p53 or may generate alternative functional proteins with oncogenic properties. The functional mutated forms of p53 protein (mutp53), including those with certain amino acid substitutions (R175, G245, R248, R249, R273, and R282), are more stable, interact with protein partners different from those of wild-type p53, and lose the original DNA-binding specificity, thus targeting genes promoting oncogenic activities such as invasion, migration, tissue remodelling, angiogenesis, stem cell expansion, proliferation, survival, and increased chemo-resistance [140,141]. Immunohistochemistry is employed in the clinical setting in order to assess the presence of mutp53 in cancer tissues, as mutp53 can easily be detected, being much more stable than the wild-type counterpart. Moreover, in several tumors including lung cancer, the accumulation of mutp53 may trigger an autoimmune response against p53 (p53Abs) [142]. In lung cancer, p53 overexpression is detected at an early stage, also in pre-neoplastic lesions, and the presence of p53 antibodies is observed many months before the lung cancer diagnosis in heavy smokers [143]. Two meta-analysis studies showed that the presence of p53 staining is an independent prognostic factor in lung cancer [144,145]. Moreover, a recent systematic review with meta-analysis showed that p53Abs are more abundant in the blood of patients affected by several types of cancers, including lung cancer, than in healthy controls [146] and together with other markers, p53Abs can be used for lung cancer detection [147]. A recent study showed that the presence of p53Abs was significantly associated with poorly differentiated and higher-grade tumors and was predictive for shorter survival in NSCLC patients [148]. A search conducted in the cBioPortal for Cancer Genomics website using data derived from The Cancer Genome Atlas [149] shows that mutp53 is present in nearly 85% of small cell lung cancer and in 60% of non-small cell lung carcinoma (Figure 3). 

All these lines of evidence indicate that mutp53 plays a pivotal role in lung tumorigenesis and can be a valid diagnostic and prognostic factor in lung cancer. Strikingly, in our laboratory it has been shown that mutp53 is able to form a complex with the transcriptional coactivator YAP and the transcription factor NFY on pro-oncogenic genes such as cyclin A, cylin B, and CDK1 [48]. This has been observed in different models of human cancers (metastatic breast cancer, head and neck and pancreatic cancer cell lines) bearing different mutp53 isoforms. Moreover, treatment with inhibitors of the mevalonate pathway reduced tumor growth in vivo and reduced the expression of mutp53/YAP oncogenic targets in vitro by impairing the recruitment of the YAP/mutp53/NFY transcriptional complex onto their promoter [48]. 

Based on these lines of evidence, it will be interesting in the future to investigate any synergism between YAP and mutp53 function in this context. Consistently, tissues from NSCLC patients with higher expression and nuclear localization of YAP showed a higher level of cyclinA [77]. In the study mentioned, the status of TP53 was not investigated, but YAP may cooperate with mutp53 on the promoter of cyclinA and other cell-cycle-related genes.

**Figure 4:**
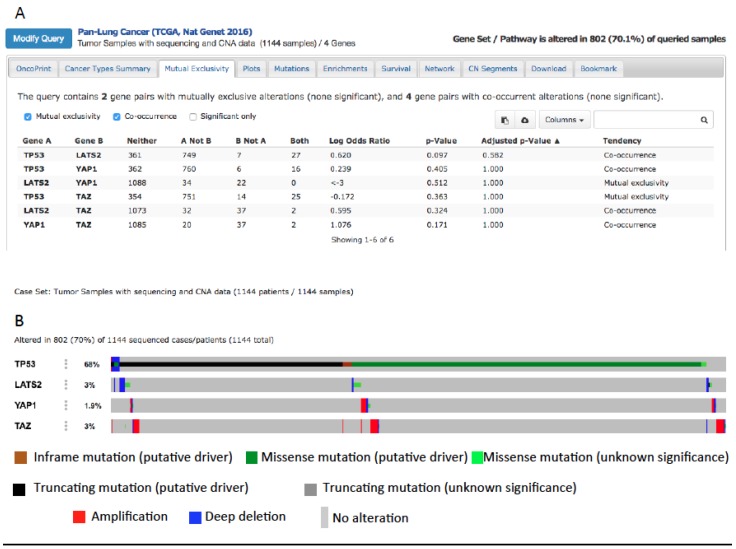
(**A**) Co-occurrence of TP53, YAP1, and LATS2 mutations in lung cancer after querying 1144 lung cancers collected in the cBioportal website database [158]. (**B**) Putative driver mutations of TP53 co-occur with YAP and TAZ amplification or with LATS2 deletion in lung cancer (from the cBioportal website).

YAP and mutp53 can synergize not only as transcriptional regulators of common oncogenic targets. In fact, mutp53 can support tumor growth both when expressed in cancer cells and also in the stroma surrounding tumors [150,151,152]. The same has been observed for YAP, which has been shown to be overexpressed in Cancer-Associated Fibroblasts (CAFs), promoting matrix stiffening and tumor growth [153]. Moreover, the Hippo and p53 signaling pathways cooperate to regulate cholesterol/lipid levels [134] which are in turn regulators of YAP. Accordingly, pharmacological inhibition of pathways involved in lipid biosynthesis inhibits YAP/TAZ oncogenic function [63,64,154,155]. In cancer cells, lipid and cholesterol biosynthesis are upregulated and drive tumor malignancy [156] partly through the upregulation of the sterol regulatory element binding protein (SREBP) transcription factors [134]. While wild-type p53 and LATS2 cooperate in inhibiting SREBP expression, mutp53 has been shown to sustain the expression of SREBP in breast cancer cells, suggesting an additional layer of positive regulation of YAP by mutp53, through the upregulation of lipid metabolism [156]. This can be investigated also in lung cancer where lipid metabolism is showing an emerging role [65,157]. Interestingly, when we interrogated 1144 samples from lung cancer patients collected in the cBioportal website database (Pan-Lung Cancer, TCGA Nat. Genet. 2016), we found that YAP amplification or LATS2 deletion co-occur with oncogenic driver mutations of the TP53 gene (Figure 4), suggesting a possible cooperation between mutp53 and the overexpression or hyperactivation of YAP in lung cancer. Interestingly, while YAP1 and TP53 alterations co-occur in lung cancer, TAZ and TP53 alterations are mutually exclusive. This is in agreement with a work from our laboratory where a transcriptional cooperation on the promoter of cell-cycle-regulating genes has been observed between mutp53 and YAP, but not between mutp53 with TAZ, highlighting a difference between YAP and TAZ [48]. Based on this observation, a detailed analysis of the TP53 mutational status combined with the activity of YAP (the latter not only determined by gene amplification but also by gene expression and protein activity) in relation to stage/grade of the tumor and prognosis of lung cancer patients can be a starting point for future studies headed in this direction.

## 7. An Emerging Role of YAP/TAZ in microRNA Regulation in NSCLC

Long lists of YAP/TAZ pro-oncogenic target genes have been published from studies on different experimental systems and conditions in order to dissect the mechanisms through which YAP/TAZ could elicit their oncogenic role [41,49,82,159,160,161,162]. However, not much is known about YAP/TAZ-regulated microRNAs, with only two reports in MCF10A cells or in human pulmonary arterial adventitial fibroblasts [163,164] and two other reports showing that YAP/TAZ can influence miRNA biogenesis by modulating the activity of specific subunits of the miRNA processing machinery [165,166] independently of YAP/TAZ function as transcriptional coactivators. 

With the exception of these studies, evidence of a direct transcriptional role of YAP/TAZ in microRNA regulation in the tumor context and on a large scale have never been investigated. This lack of knowledge is relevant, since altered miRNA expression can contribute to tumorigenesis by inappropriately modulating critical genes involved in tumor cell biology. Our recent study is the first report of a list of microRNAs specifically regulated by YAP and TAZ in NSCLC [135]. We focused on a cluster of three oncogenic microRNAs (miR-25, 93, and 106b) located in the intron 13 of MCM7 (Minichromosome Maintenance complex 7) gene. Both the coding MCM7 transcript and the hosted microRNAs are oncogenic, and they are upregulated in lung cancer tissues and show a prognostic role [167,168,169,170,171,172,173]. We found that MCM7 transcript and the hosted microRNAs form a bi-oncogenic locus [174] that is positively regulated by YAP/TAZ as a single transcriptional unit [135]. We showed that in NSCLC cell lines, MCM7 and hosted miRs repress a list of cancer-related genes, and focused on the p21 cell cycle inhibitor. Two previous works showed YAP as a negative regulator of the p21 gene at a transcriptional level [175,176], while we elucidated a new layer of p21 inhibition mediated by YAP/TAZ through a post-transcriptional mechanism [135]. Moreover, these findings hold great potential for translational medicine as microRNAs are frequently used as molecular biomarkers of diagnostic and prognostic relevance in human cancers, for patient stratification and for monitoring the response of cancer patients to treatment. It will be important in the future to characterize the whole transcriptome under YAP/TAZ modulation in addition to microRNAs because competing endogenous RNAs, long noncoding RNAs, and enhancer-associated RNAs are emerging for their role in development and cancer, including lung cancer [177,178]). In agreement with this hypothesis, YAP/TAZ have recently been globally mapped at enhancers much more than in the proximity of transcription start sites [49].

## 8. YAP/TAZ as Therapeutic Targets in NSCLC: State of the Art

Current therapy for lung cancer involves platinum-based chemotherapy, while radiation is used for the treatment of locally advanced disease, concurrently with platinum chemotherapy. These therapies, however, show strong side effects in patients because they are highly cytotoxic also for healthy cells. In order to overcome these side effects, increased effort is being put towards targeted therapy according to the biomarker status of the tumor. The best characterized driver mutations for lung cancer are KRAS, EGFR, ALK, ROS1, BRAF, and MET. These mutations induce upregulation of hyperactivation of the encoded receptors or kinases belonging to different signalings (RAS/RAF/MEK/ERK, JAK/STAT, SRC/STAT, PI3K/AKT/mTOR) which are responsible for the aberrant proliferation and survival of cancer cells. Thus, inhibition of these signaling pathways through employing small molecule inhibitors of kinases or monoclonal antibodies against receptors may be a good therapy for patients whose driver oncogenic mutations are known. Antagonists of the epidermal growth factor receptor (EGFR), ALK-ROS1 inhibitors, and RAF-MEK inhibitors have been successfully used in NSCLC clinical trials and approved by the FDA, while the employment of KRAS inhibitors in clinics needs further trials [179,180]. Promising results have been obtained with EGFR inhibitors, which after several randomized phase 3 trials have been approved as first-line treatment in advanced NSCLC patients positive for EGFR mutations, showing higher progression-free survival and lower toxicity when compared to standard chemotherapy [181]. However, in Western populations, only 20% of patients with metastatic lung adenocarcinoma can be stratified for treatment with current targeted therapies with median responses of approximately a year [182,183]; thus, cytotoxic chemotherapy remains the elected therapy for first-line treatment of advanced disease. Often, the emergence of resistance to chemotherapy, radiotherapy, or targeted therapies reduces their efficacy after an initial response [184,185,186]. Resistance to therapies may involve the acquisition of new specific mutations by cancer cells that re-activate the signaling pathways targeted by the therapy through some alternative mechanisms. Thus, it is important to dissect the molecular mechanisms regulating not only lung tumor formation, progression, and metastasization, but also the mechanisms responsible for the primary and acquired resistance to therapy of cancer cells in order to overcome them, for example, by inhibiting the bypass pathways conferring resistance. Interestingly, as discussed below, all the driver genes for lung cancer listed above along with their downstream pathways have been shown to regulate YAP and TAZ at different levels in several contexts, including lung cancer. Thus, inhibition of YAP and TAZ downstream of these pathways may provide a valid therapeutic opportunity in addition to current targeted therapies. 

The clinical targeting of YAP and TAZ in cancer is attractive because in most adult organs, and in the absence of any injury, they seem to be unnecessary for normal tissue homeostasis, while they appear to be required for tumor survival, growth, metastasization, and resistance to several treatments. Thus, their inhibition may have beneficial effects on cancer therapy without gross side effects on normal cells and healthy tissues. In line with this, several recent reports provided evidence that YAP and TAZ pharmacological inhibition reduces their oncogenic function in NSCLC models (see below), and, importantly, synergizes with current treatments which inhibit the main oncogenic pathways driving NSCLC (see Section 9).

A recent study showed that norcantharidin (NCTD) inhibits cell growth, migration, and invasiveness while enhancing apoptosis and senescence in NSCLC cells, partly through a specific downregulation of an aberrantly activated YAP signaling observed both in NSCLC cell lines and tumor tissues [76]. NCTD was already applied in treatment of breast cancer, liver cancer, gallbladder carcinoma, prostate cancer, mantle cell lymphoma, hepatocellular carcinoma, leukemia, and colon cancer with few side effects [187,188,189,190,191,192,193,194,195]. The study by Guo et al. shows the NCTD role in the inhibition of YAP-driven oncogenic pathways and suggests the possibility to also use this drug in lung cancer therapy. Another recent work showed that rottlerin, a natural polyphenolic compound derived from *Mallotus philipinensis*, exhibits antitumor activity in NSCLC cells (inhibits cancer cell proliferation, migration, and invasion, and enhances cell cycle arrest and apoptosis) partly through the inhibition of oncogenic TAZ [196], supporting the intriguing possibility to use natural compounds in order to inhibit oncogenic YAP and/or TAZ function. Other possible therapies against oncogenic YAP and TAZ may be represented by compounds able to activate their endogenous inhibitors. For example, LKB1, which inhibits YAP and TAZ in response to energy stress [84,122], has been shown to be induced and activated by Honokiol (HNK), a bioactive molecule from *Magnolia grandiflora*, in breast cancer experimental models [197]. Honokiol was able to inhibit breast tumorigenesis in mice in an LKB1-dependent manner [197]. Honokiol antitumor effects have been experimentally tested in different types of cancer, including lung cancer, where it was shown to play an anti-metastatic role through the inhibition of pro-metastatic proteins, among which was β-catenin, known to synergize with YAP/TAZ and to be a target itself of YAP and TAZ [198,199,200]. Finally, the following GPCRs (GPR87, CMKOR1, LGR4, FZD10, and P2RY11) were observed to be overexpressed in NSCLC patients [201] and some of their natural ligands were shown to activate oncogenic YAP and TAZ function [202]. This suggests that pharmacological inhibition of these GPCRs could represent another mechanism through which the pro-oncogenic function of YAP and TAZ can be inhibited in NSCLC [203]. All these observations need to be translated into clinics.

## 9. Synergism between YAP/TAZ Inhibition and Current Therapies in NSCLC Experimental Models

Recent studies have shown that in NSCLC, the hyperactivation of YAP may confer increased patient resistance to several treatments including cisplatin, radiation therapy, EGFR inhibitors, and BRAF or MEK inhibitors [102,136,137,138,204]. 

In those studies, YAP was overexpressed in cells that acquired resistance to the mentioned therapies, while knock-down of YAP and TAZ or treatment with the YAP/TAZ pharmacologic inhibitor verteporfin enhanced the response to cisplatin, radiation therapy, and EGFR inhibitors [136,137,138,204]. The mechanisms at the basis of resistance can be multiple and are described below.

Chemo-radiotherapy: Platinum-based chemotherapeutic drugs crosslink with the purine bases on the DNA, interfering with DNA repair mechanisms, eventually causing DNA damage, and inducing apoptosis in cancer cells. Pemetrexed inhibits purine and pyrimidine synthesis, interfering with cell proliferation and DNA damage repair, while radiotherapy causes DNA damage and cell death via the generation of free radicals. It has been proposed that YAP and TAZ may be involved in the protection of genomic stability in different models, including lung cancer [64,205,206] and that YAP stimulates nucleotide biosynthesis in liver cancer [207]. This suggests that YAP- and TAZ-overexpressing cells can overcome the DNA damage and the inhibition of DNA synthesis induced by chemotherapy. YAP and TAZ can also reduce sensitivity to DNA damaging agents by upregulating signaling which bypass cell cycle checkpoints induced by DNA damage, or by upregulating pro-survival and pro-proliferative signaling, typically hyperactivated in cells with stemness properties, where YAP/TAZ are often overexpressed and whose number increases over the course of radiotherapy and chemotherapy to repopulate the tumor mass [208]. It has also been shown that hyperactivation of β-catenin signaling can increase chemoresistance and radioresistance in different cancer types [209,210,211], and a synergism between β-catenin and YAP/TAZ has been described in cancer, including lung cancer [64,212,213,214], suggesting a possible role of YAP and TAZ in chemoresistance and radioresistance through its crosstalk with β-catenin signaling that needs to be further investigated in lung cancer. All the mechanisms described above can explain how cancer cells overexpressing YAP and/or TAZ can bypass the inhibitory effect of cytotoxic chemotherapy and radiotherapy.

Resistance to EGFR inhibitors: EGFR receptors, activated by the EGF ligands, induce three main pro-survival signaling pathways: SRC/JAK/STAT signaling, RAS/RAF/MEK/ERK signaling, and PI3K/AKT/mTOR signaling.

YAP has been shown to be overexpressed and more active in NSCLC specimens bearing higher resistance to EGFR inhibitors, and to mediate this resistance through several mechanisms [102,136,204,215]. One study shows that YAP overexpression increases Erbb3 [204]; through the activation of the Erbb3 signaling, NSCLC cells can reactivate ERK1/2 also in the presence of EGFR inhibitors [216,217]. In another study, YAP has been shown to activate AXL tyrosine kinase which induces PI3K/AKT signaling, providing a parallel survival input in the presence of EGFR inhibitors [215,218]. Finally, a recent study has shown that YAP works in parallel with RAF and MEK in the regulation of the antiapoptotic gene BCL-xL, thus promoting resistance to RAF and MEK inhibitors in BRAF mutant NSCLC.

In this scenario, YAP/TAZ inhibition may have a therapeutic effect when combined with other therapies already used for lung cancer treatment. Strikingly, in the abovementioned studies, YAP is pharmacologically inhibited with verteporfin, which disrupts its interaction with the TEAD transcription factor. This is important because verteporfin is already used in clinics for neovascular macular degeneration and opens up the opportunity to also use this drug in improving the response to current treatments in lung cancer.

**Figure 5:**
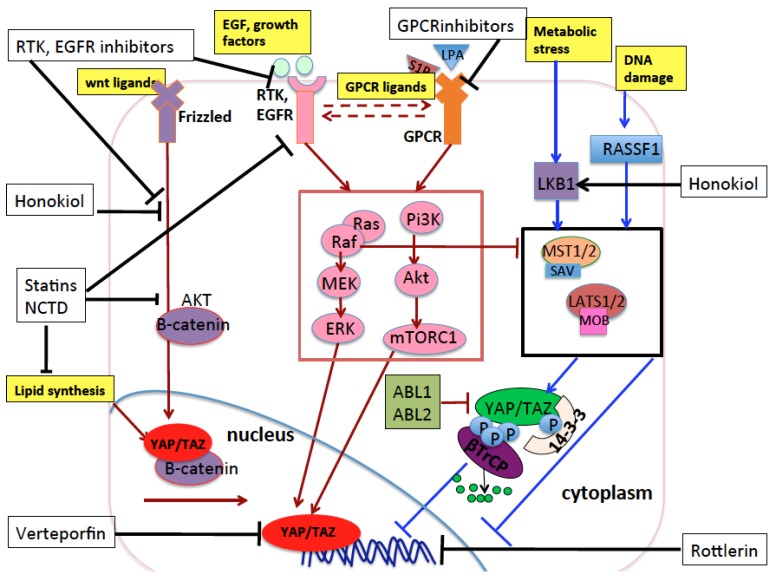
Schematic representation of the pharmacological compounds (black boxes) that inhibit YAP and TAZ oncogenic function through targeting YAP and TAZ or the main proteins and pathways that influence YAP and TAZ in lung cancer. Honokiol reactivates LKB1 and inhibits the YAP/TAZ/β-catenin oncogenic pathway. Statins and norcantharidin (NCTD) inhibit the mevalonate pathway and the production of monounsaturated fatty acids—two biosynthetic pathways of lipids that are increased in tumorigenesis. Through the inhibition of these two pathways, statins and norcantharidin inhibit oncogenic YAP, TAZ, and β-catenin signaling. Statins also inhibit EGFR autophosphorylation and β-catenin activation and nuclear translocation. Rottlerin is a natural polyphenolic compound which inhibits oncogenic TAZ in lung. EGFR inhibitors are too many to be listed here, but they inhibit the EGFR receptors and their downstream effectors and their inhibition has a synergistic effect when combined with YAP inhibition mediated by Verteporfin. This latter impairs the binding of YAP with the oncogenic transcription factors TEADs. Black arrows indicate pharmacological activation of the targeted proteins or pathways. Black blunt lines indicate pharmacological inhibition of the targeted proteins or pathways. As in previous figures, lines or arrows in red indicate signals which eventually activate nuclear YAP/TAZ, while lines or arrows in blue indicate signals or proteins which eventually inhibit nuclear YAP/TAZ, through mechanisms either dependent on or independent of Hippo pathway core kinases. Dashed arrows indicate reciprocal crosstalk between receptors and their downstream transduction.

Growing evidence shows that statins—inhibitors of the mevalonate (MVA) pathway, conventionally used in the treatment of hypercholesterolemia in cardiovascular disorders—also inhibit YAP-/TAZ-mediated transcriptional activation of pro-oncogenic genes and reduce breast tumor growth in mice xenografts [48,63,155]. Statins and similar compounds showed a promising role in the prevention and treatment of different cancer types in some preclinical and epidemiological studies, while in other studies the therapeutic efficacy and safety of statin was questionable (reviewed in [219]). Moreover, evidence of a therapeutic role of statin in lung cancer is still lacking. In vitro, we previously showed that treatment of NSCLC cells with the statin cerivastatin partially recapitulated the tumor suppressive effects observed upon YAP/TAZ interference, while treatment with MVA reversed the cerivastatin effect [135]. This result reinforces previous experimental works where statins showed anticancer effects in NSCLC cell lines [220,221,222] and in mouse models of lung cancer [222], linking the anti-oncogenic potential of statins to the inhibition of YAP and TAZ. At a clinical level, two recent prospective studies on a large population-based cohort of lung cancer patients showed that patients who used statins prior to the cancer diagnosis had reduced rates of cancer-specific mortality [223,224] and provided some evidence (to be confirmed with clinical trials) that statins may increase the survival of patients when prescribed at the time of lung cancer diagnosis [224]. A more recent prospective study showed that statin treatment increased survival in a large nationwide (Taiwan) population-based cohort of patients with lung cancer concomitantly receiving EGFR-TKI therapy [225], in line with previous case-control studies [226]. At a molecular level, statins were shown to act synergistically with conventional EGFR inhibitors in models of breast cancer and head and neck squamous cell carcinomas (HNSCC) through the inhibition of EGFR autophosphorylation [227]. In models of NSCLC, statins counteract the resistance to EGFR-TKI through the suppression of AKT/β-catenin signaling [228]. Moreover, as mentioned above, statins inhibit the oncogenic function of YAP, and YAP inhibition increases sensitivity to EGFR-TKI. Overall, these studies expand the potential therapeutic application of inhibitors of the mevalonate pathway in NSCLC, where early-phase clinical trials are still ongoing employing statins in combination with the current therapies (ClinicalTrials.gov Identifier: NCT00966472, NCT00452634, NCT00433498, NCT00452244, NCT01441349 (see the website http://clinicaltrials.gov)). All the possible therapeutic strategies targeting oncogenic YAP and TAZ and their upstream regulators are schematically represented in Figure 5. 

## 10. Immune Evasion

Cancer cells often acquire the ability to evade the host immune response, even in the presence of an intact immune system, through multiple mechanisms. The dissection of the mechanisms driving immune evasion is under investigation with the aim to make the tumor more sensitive to the host immune response [229]. Several lines of evidence have suggested that YAP can be involved in acquired immune tolerance in different types of cancer cells. For example, in murine models of prostate cancer, YAP1 has been shown to promote the transcription of Cxcl5, a chemochine which attracts myeloid-derived suppressor cells (MDSCs) to the tumor microenvironment, reducing the immune anti-tumor response, and the knock-down of YAP1 reduced MDSCs in the intratumoral population of immune cells, impairing tumor progression [230]. The increased presence of MDSCs has been observed in patients with various solid tumors, including lung cancer, where a higher number of MDSCs correlates with tumor progression and poor prognosis [231,232,233]. Promising preclinical studies showed that pharmacological inhibition of MDSCs contributes to reducing tumor growth, progression, and metastasis, and increases the response to chemotherapy and radiotherapy in lung cancer models. However, these studies need to be translated into clinics (reviewed in [234]). Another study showed that in mouse epicardial cells, YAP and TAZ recruit a specific subset of suppressive CD4^+^ Tregs to injured myocardium after myocardial infarction. However, functional evidence of a role of YAP and TAZ in immunosuppression in vivo in lung cancer is still scarce. Two recent studies have shown that YAP transcriptionally upregulates PD-L1 (programmed cell death-ligand 1) in NSCLC cells [235,236]. PD-L1 is a ligand which binds to the PD1 receptor, an immune checkpoint receptor involved in tumor immune escape [237], and its overexpression is prognostic in lung cancer and increases cancer cell proliferation and chemoresistance [238,239]. PD-L1 has been widely studied in the few last years as a novel therapeutic target in lung cancer patients, and PD1/PD-L1 inhibitors are employed in several clinical trials in comparison or in combination with current therapies. 

In August 2017, in a randomized, pivotal, phase 3 study of KEYNOTE-024 (ClinicalTrials.gov Identifier: NCT02142738), pembrolizumab, a PD1 inhibitor, was approved by FDA as a first-line therapy for advanced metastatic (stage IV) NSCLC, according to PD-L1 expression status of the tumor, giving superior progression-free survival in comparison to platinum-based therapy. Importantly, the 305 enrolled patients were found positive to programmed cell death ligand 1 (PD-L1) (in 50% of tumor cells or more) and lacked any EGFR or ALK aberrations. In another recent phase III clinical trial (Pacific), the PD-L1 inhibitor durvalumab (Imfinzi) was approved by FDA for the treatment of patients with locally advanced, unresectable stage III NSCLC who had not progressed following chemoradiotherapy. In that trial (where 713 patients were enrolled), durvalumab improved the median progression-free survival by 11.2 months compared to placebo, irrespective of PDL1 expression before chemoradiotherapy (ClinicalTrials.gov Identifier: NCT02125461). Other clinical trials are still ongoing for employing a PD-L1 blockade in combination with stereotactic radiation therapy (SRT) in stage I–IIIA NSCLC patients who are planned to undergo surgical resection of lung cancer (ClinicalTrials.gov Identifier: NCT03217071) or in combination with standard chemotherapy or EGFR-targeted therapies. Future studies are needed in order to demonstrate any possible role of YAP and TAZ in the regulation of immune evasion in human lung cancer through PDL1, Cxcl5, or other mechanisms. 

## 11. Concluding Remarks

The Hippo pathway regulates several physiological processes and is implicated in numerous diseases including cancer. This makes it an exciting field of investigation. Knowledge on the mechanisms regulating and regulated by this pathway is continuously expanding, opening a wide range of possible players which can be therapeutically targeted in clinics. The oncogenic role of YAP and TAZ in lung cancer has been widely demonstrated. 

However, in general, YAP and/or TAZ activation alone is not sufficient to drive tumorigenesis, but it promotes tumor growth and aggressiveness when it occurs concurrently with other oncogenic modifications of tumor cells and/or the tumor microenvironment, which may vary in different contexts. Moreover, it is important to keep in mind that YAP and TAZ, even if paralogues, can exert completely different, and sometimes opposite, functions in the same context or tissue. For example, in contrast to what has been widely shown for YAP (increased YAP expression and activation in NSCLC specimens resistant to EGFR-targeted therapy), Noguchi and coworkers showed that TAZ is overexpressed in cells that are more sensitive to EGFR inhibitors [80]. Thus, further knowledge is still required in order to understand controversial experimental findings and to design the correct therapy for each case, when combining current therapies with inhibitors of YAP and/or TAZ oncogenic activity in YAP- and /or TAZ-positive lung cancer specimens, in the different genetic and epigenetic backgrounds of lung cancer.

## Figures and Tables

**Table 1:** Hippo pathway components and upstream regulators found to be dysregulated in non-small cell lung cancer (NSCLC) in previous experimental reports.

Hippo Pathway Component/ Regulator	Increase/Decrease/Prognosis	Reference	N
YAP	Protein level increases in 70% NSCLC tissues. Bad prognosis. Correlation with late T stage, TNM stage and lymph node metastasis.	Su, L.L., et al.,2012 [72]	40
	Protein level increased in 66.3% NSCLC tissues and predominantly nuclear. Bad prognosis. Correlation with TNM stage, lymph node metastasis, shorter O.S.	Wang, Y., et al.,2010 [73]	92
	YAP gene amplified in NSCLC cell lines and in 23% NSCLC tissues.	Lorenzetto E., et al, 2014 [74]	77
	Protein Level and Nuclear localization Increased in LAC. Bad prognosis. correlation with TNM stage, cyclinA overexpression, increased EGFR copy number.	Kim, J.M., et al., 2011 [75]	168
	Increased Nuclear localization. Increased nuclear and reduced cytoplasmic expression in NSCLC cell lines and tissues.	Guo, J., et al., 2017 [76]	4
	Protein level increased in 87.8% LAC. Bad prognosis.	Cui, Z.L., et al., 2012 [77]	49
	YAP1 R331W Missense germline mutation in 1.1% patients with LAC with respect to 0.18% in healthy control. Predictive of LAC predisposition.	Chen, H.Y., et al.,2015 [81]	1312 LAC 1135 healthy controls
	Bad prognosis. Shorter survival when overexpressed with TAZ, SCD1 and β-catenin.	Noto, A., et al., 2017 [64]	10
TAZ	Bad prognosis. Shorter survival when overexpressed with YAP, SCD1 and β-catenin.	Noto, A., et al., 2017 [64]	10
	Overexpressed in NSCLC cells.	Zhou, Z., et al., 2011 [78]	cells
	Overexpressed in 66.8% NSCLC. Bad prognosis. Correlated with TNM stage and lymph node metastasis, invasion, shorter O.S and D.F.S.	Xie, M., et al., 2012 [79]	181
	Bad prognosis. Shorter survival. High TAZ expression at the genomic, mRNA, and protein levels in Squamous cell Carcinoma patients with respect to controls.	Noguchi, S., et al., 2014 [80]	345 NSCLC patients 18 healthy controls
LATS2	Transcript Downregulated in NSCLC.	Strazisar, M., et al., 2009 [103]	129
	Promoter hypermethylated in 71% NSCLC.	Malik, S.A., et al., 2017 [105]	79
	Lower protein and trasnscript expression in LAC. Good prognosis. Increased O.S and D.F.S.	Luo, S., et al., 2014 [108]	79
LATS1	Promoter hypermethylated in 66.66% NSCLC.	Malik, S.A., et al., 2017 [105]	79
	Good prognosis. correlated with TNM stage, lymph node metastasis.	Lin, X.Y., et al., 2014 [109]	136
AMOT	Downregulated in 45.8% NSCLC patients	Hsu, Y.L., et al., 2014 [111]	24
ABL1, ABL2	ABL1 and 2 are somatically mutated in 1.5% and 4% NSCLC patients, ABL2 is amplified in 8% NSCLC.	Testoni, E., et al., 2016 [88]	N.A
LKB1	Gene mutated in 18% LAC patients.	Ding, L., et al., 2008 [124]	188
	Gene inactivated in 54% of LAC cell lines.	Carretero, J., et al., 2004 [125]	11
	Gene inactivated in 33% NSCLC patients.	Sanchez-Cespedes, M., et al., 2002 [127]	20
	Gene mutated in 8% NSCLC patients (7/91) and in 39% (20/51) NSCLC cell lines. Good prognosis.	Matsumoto, S., et al., 2007 [128]	91 patients 51 cell lines
RASSF1A	Promoter hypermethylated in 40% of primary lung tumors, missense mutation in 10% of primary lung tumors.	Dammann, R., et al., 2000 [117]	60
	Promoter hypermethylated in 72% of SCLC, 34% of NSCLC. Loss of heterozigosity (LOH) in 86% SCLC (31/36), 91% (10/11) Squamous cell carcinoma, 71% (15/21) LAC.	Agathanggelou, A., et al., 2001 [118]	29 SCLC, 41 NSCLC (hypermet study) 36 SCLC, 48 NSCLC (LOH study)
	Transcript Not expressed in 100% SCLC, 64% NSCLC. Promoter hypermethylated in 100% SCLC. Good prognosis. Hypermethylation was associated with impaired patient survival.	Burbee, D.G., et al., 2001 [119]	47 SCLC, 107 NSCLC
	Promoter hypermethylated in 21.8% NSCLC patients. Good prognosis in patients expressing higher levels of RASSF1A.	de Fraipont, F., et al., 2012 [120]	202
VGLL4	Transcript downregulated in LAC tumor respect to non tumoral tissues (29/30) Lower levels of nuclear protein in LAC compared to normal lungs: 92.6% of patients (25/27) exhibited high nuclear VGLL4 expression in their normal lungs, whereas only 22.1% of patients (17 out of 77) had high nuclear expression of VGLL4 in their lung ADCs	Zhang, W., et al., 2014 [113]	30
Ski	Promoter hypermethylated in 56% of NSCLC tumor samples and in NSCLC cell lines Good prognosis.	Xie, M., et al., 2017 [130]	168

Red indicates proteins that activate YAP or TAZ oncogenic function, are upregulated or hyperactivated in NSCLC, and whose upregulation is associated with poorer prognosis in NSCLC. Blue indicates proteins that inhibit YAP and TAZ oncogenic function, are downregulated in NSCLC and whose upregulation is associated with better prognosis. When control patients are not indicated, number of patients indicates only those who bear NSCLC. When the number of control patients is indicated, the study compares NSCLC patients with healthy controls. LAC = lung adenocarcinoma; TNM = tumor node metastasis stage; D.F.S. = disease-free survival; O.S. = overall survival; N = number of patients or cell lines.
